# Rubber Band Ligation for Treatment of Mucoceles: A Potential Minimally Invasive Treatment Approach

**DOI:** 10.3390/reports9030281

**Published:** 2026-08-25

**Authors:** Satoru Kusaka, Tatsuya Akitomo, Masashi Ogawa, Yuto Shoji, Jimei Zhao, Ryota Nomura

**Affiliations:** 1 Department of Pediatric Dentistry, School of Dentistry, Osaka Dental University, Osaka 573-1121, Japan; 2 Department of Pediatric Dentistry, Hiroshima University Hospital, Hiroshima 734-8551, Japan; 3 Department of Pediatric Dentistry, Graduate School of Biomedical and Health Sciences, Hiroshima University, Hiroshima 734-8553, Japan

**Keywords:** mucocele, pediatric dentistry, pediatric patient, case report

## Abstract

Mucoceles are benign lesions caused by the accumulation of mucus such as saliva, and are common in children. Surgical intervention is effective but is not well tolerated by children because of its invasiveness, and alternative treatment methods are being explored. A boy aged 2 years and 5 months presented at our hospital with a chief complaint of mucocele. Considering the patient’s age, micro-marsupialization was initially selected to avoid surgical excision; however, the cyst did not resolve. We decided to apply the rubber band ligation method used for treatment of internal hemorrhoids, and ligated the mucocele using an orthodontic elastic separator. At the 3-week follow-up visit, the mucocele had disappeared. According to the patient’s guardian, the rubber band fell off after 1 week, and the mucocele subsequently detached and fell off spontaneously shortly thereafter. Although further studies are needed to establish the effectiveness of this treatment method, this report suggests that rubber band ligation may represent a novel, minimally invasive, and safe therapeutic option for the treatment of mucoceles.

**Figure 1:**
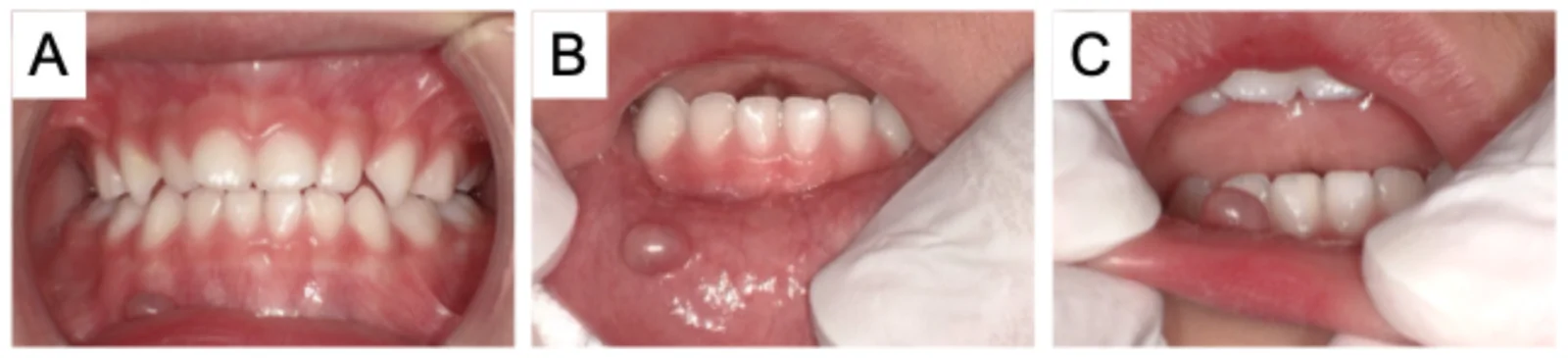
Intraoral photographs at the initial visit (**A**) during occlusion, (**B**) during rotation, (**C**) lateral view. A boy aged 2 years and 5 months was referred to our hospital with a mucocele. A bulge in the lower lip had been noted at the patient’s 18-month checkup, and he had visited a private dental clinic. The lesion underwent repeated episodes of slight swelling and regression, but did not improve, and the patient was then referred to our hospital. A small, bean-sized swelling was noted on the right lower lip. The lesion was soft and elastic, with palpable fluctuance. There were no other abnormal findings in the oral cavity, and there was no relevant family or medical history. Based on the clinical findings and the lesion’s history, a diagnosis of mucocele was confirmed.

**Figure 2:**
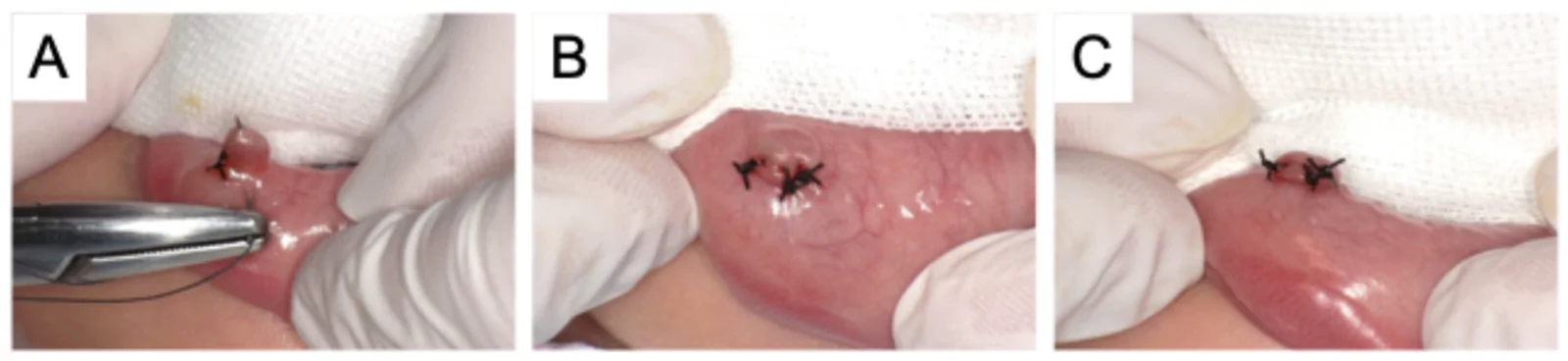
Intraoral photographs during micro-marsupialization (**A**) during suturing, (**B**) during rotation, (**C**) lateral view. Because the patient was only 2 years old, micro-marsupialization was our first treatment choice [1]. Following local anesthesia, three silk sutures were passed through the cystic cavity, and each suture was tied over the cyst wall. Perforation of the cyst resulted in slight shrinkage of the lesion by the end of the procedure.

**Figure 3:**
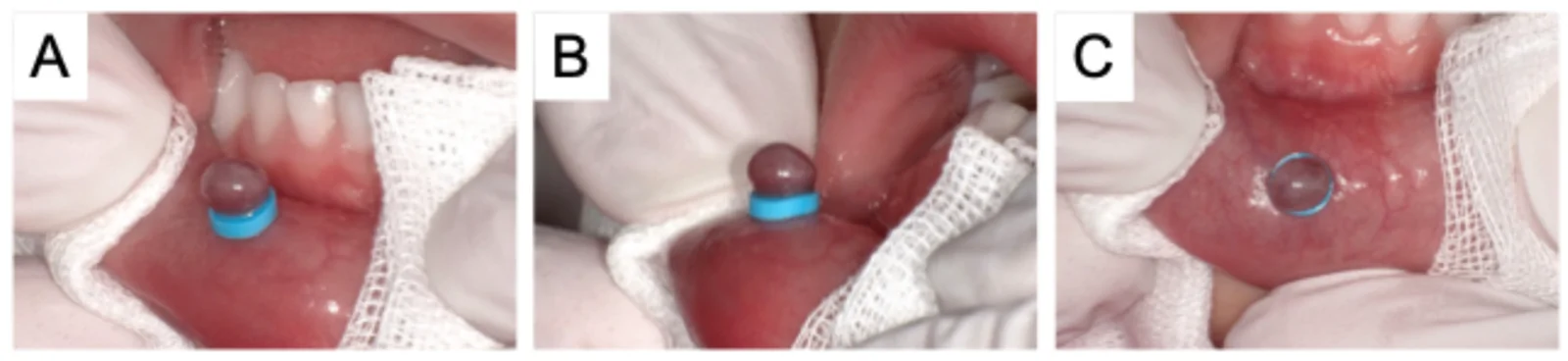
Intraoral photographs after rubber band ligation (**A**) during rotation, (**B**) lateral view, (**C**) frontal view. A follow-up examination 2 weeks after the micro-marsupialization revealed that the sutures had fallen out prematurely and there was no change in the mucocele. Informed consent was then obtained from the patient’s guardians to use rubber band ligation. An orthodontic elastic separator measuring 5 mm in outer diameter and 2 mm in inner diameter was selected based on the size of the cyst. Without administering local anesthesia, the separator was applied to the base of the cyst using orthodontic separating pliers. The patient experienced no pain or discomfort after the ligation.

**Figure 4:**
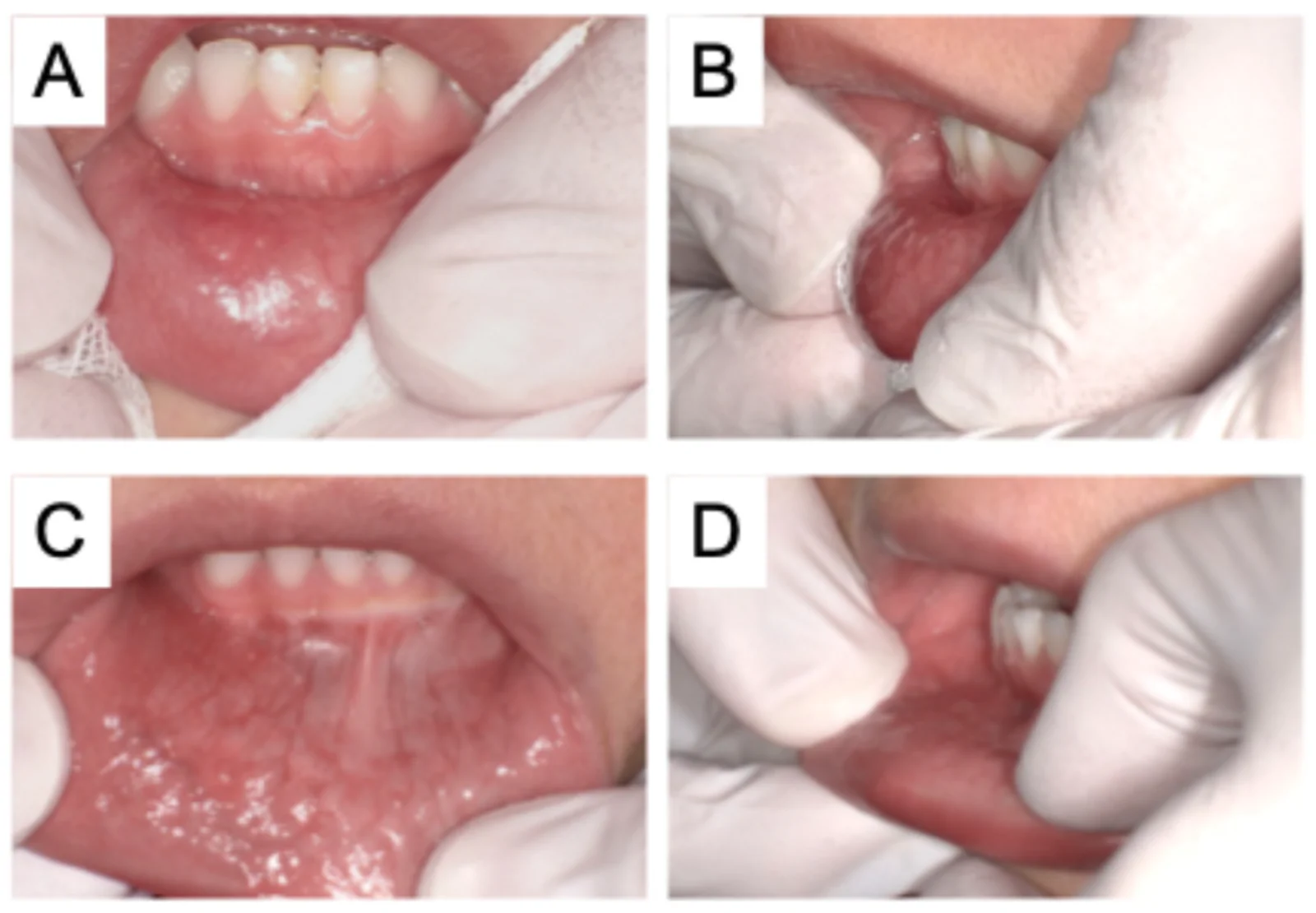
Intraoral photographs in the follow-up period. At the 3-week follow-up, neither the mucocele nor the rubber band was present in the oral cavity (**A**,**B**). The guardian reported that the rubber band detached spontaneously approximately 1 week after treatment, followed by spontaneous sloughing off of the mucocele. At the 3-month follow-up appointment, there was no recurrence of the mucocele (**C**,**D**), and the patient was discharged into the care of a private dental clinic. A mucocele is a benign lesion caused by the accumulation of mucus inside the tissues, and appears on the mucosa as a soft, well-defined, painless swelling [2]. It affects both sexes, with peak incidence among children and young adults [3]. The most effective treatment for mucoceles is surgical excision, which can be challenging in children and patients with behavioral problems [3,4]. Therefore, non-invasive methods for treating mucocele have been sought, including laser ablation, electrosurgery, micro-marsupialization, and cryosurgery [5]. Laser ablation and electrosurgery cause less bleeding and pain compared with conventional scalpel surgery [5]. On the other hand, Tsunoda et al. (2021) evaluated the effectiveness of cryotherapy for mucoceles and reported that it is a convenient and safe therapeutic approach for preschool-aged children [6]. However, performing these procedures requires specialized instruments and devices, such as a freezing device. Therefore, in the present case, we initially selected micro-marsupialization; however, the sutures fell out prematurely, and the cyst did not improve. Although micro-marsupialization is intended to minimize patient invasiveness, it is not a definitive treatment, and complete resolution of the mucocele was not achieved. Reducing invasiveness to the patient presents the challenge of an increased risk of recurrence, highlighting the need for a safe and simple treatment approach that can be applied even in young children.

Rubber band ligation is a common treatment for internal hemorrhoids, and ligation of the hemorrhoidal tissue with a rubber band causes ischemic necrosis and scarring, leading to fixation of the connective tissue to the rectal wall [7,8]. The contents of a mucocele are mainly saliva, and because necrosis caused by ligation does not need to be considered, we applied rubber band ligation to the mucocele. In patients undergoing rubber band ligation for hemorrhoids, complications such as pain and discomfort have been reported; however, no complications were observed in the present case [8]. The rubber band fell off after approximately 1 week, and the mucocele healed naturally. In addition, no pain was reported at the time of detachment, and no recurrence was observed after 3 months of follow-up. The detachment of the mucocele may have resulted from ischemic necrosis, as observed in the process of detachment of internal hemorrhoids. However, the possibility that the lesion underwent spontaneous rupture or regression due to alterations in internal pressure induced by ligation cannot be excluded. Further investigation is needed to elucidate the mechanism underlying this treatment approach. Because this treatment does not involve a surgical procedure, it does not require anesthesia. Furthermore, there is no risk of procedure-related complications caused by patient movement during treatment. These features indicate a high level of safety, particularly for younger patients, and represent a unique advantage over other treatment modalities.

This report has some limitations. First, this is a single case report, and further studies with a larger number of cases are needed to determine whether rubber band ligation is an effective treatment for mucoceles. Additionally, its application is limited to selected cases because only mucoceles of a certain size and thickness are suitable for rubber band ligation. Because the rubber band has the potential to detach spontaneously, there is a risk of accidental ingestion or aspiration. In this case, the lesion was located slightly outside of the lower lip, where parents could easily monitor it; however, the indication for this technique should be carefully considered depending on the location of the mucocele. Although there are many limitations, this report suggests that rubber band ligation has potential as a minimally invasive and safe treatment option for mucoceles.

## Data Availability

The original contributions presented in this study are included in the article. Further inquiries can be directed to the corresponding author.
